# Population Dynamics of Wide Compatibility System and Evaluation of Intersubspecific Hybrids by *indica*-*japonica* Hybridization in Rice

**DOI:** 10.3390/plants11151930

**Published:** 2022-07-26

**Authors:** Jayanth Kallugudi, Vikram Jeet Singh, Kunnumal Kurungara Vinod, Subbaiyan Gopala Krishnan, Shekharappa Nandakumar, Brijesh Kumar Dixit, Ranjith Kumar Ellur, Haritha Bollinedi, Mariappan Nagarajan, Amit Kumar, Mridul Chakraborti, Rakesh Kumar Seth, Tapan Kumar Mondal, Prolay Kumar Bhowmick, Ashok Kumar Singh

**Affiliations:** 1 Division of Genetics, ICAR-Indian Agricultural Research Institute, New Delhi 110012, India; jakjayanth@gmail.com (J.K.); jeet2012vikram@gmail.com (V.J.S.); kkvinod@iari.res.in (K.K.V.); gopal.s@icar.gov.in (S.G.K.); nandus237@gmail.com (S.N.); brijeshdixit152@gmail.com (B.K.D.); ranjith.ellur@icar.gov.in (R.K.E.); haritha.bollinedi@icar.gov.in (H.B.); director@iari.res.in (A.K.S.); 2 Rice Breeding and Genetic Research Centre, ICAR–Indian Agricultural Research Institute, Aduthurai 612101, India; m.nagarajan@icar.gov.in; 3 ICAR–Research Complex for North Eastern Hill Region, Umiam 793103, India; amit.kumar3@icar.gov.in; 4 Division of Crop Improvement, ICAR–National Rice Research Institute, Cuttack 753006, India; mridul.chakraborti@icar.gov.in; 5 ICAR–Indian Agricultural Research Institute, Regional Station, Karnal 132001, India; rakesh.seth@icar.gov.in; 6 ICAR–National Institute of Plant Biotechnology, New Delhi 110012, India; tapan.mondal@icar.gov.in

**Keywords:** *indica-japonica*, wide compatible varieties, *S5n*, inter sub-species hybridization

## Abstract

The exploitation of heterosis through intersubspecific hybridisation between *indica* and *japonica* has been a major breeding target in rice, but is marred by the cross incompatibility between the genomes. Wide compatibility (WC) is a triallelic system at the *S5* locus on chromosome 6 that ensures the specificity of hybridisation within and between *indica* and *japonica.* The *S5n* allele that favours intercrossing is sparsely distributed in the rice gene pool and therefore warrants identification of diverse WC sources to develop superior intersubspecific hybrids. In this study, we have identified several novel WC sources through the marker-assisted screening of a large set of 950 rice genotypes. Seventeen percent of the genotypes carried *S5n*, which fell into two subpopulations. The WC genotypes showed wide phenotypic and genotypic variability, including both *indica* and *japonica* lines. Based on phenotypic performance, the WC varieties were grouped into three clusters. A subset of 41 WC varieties was used to develop 164 hybrids, of which WC/*japonica* hybrids showed relative superiority over WC/*indica* hybrids. The multilocation evaluation of hybrids indicated that hybrids derived from WC varieties, such as IRG137, IRG143, OYR128, and IRGC10658, were higher yielding across all the three different locations. Most of the hybrids showed the stability of performance across locations. The identified diverse set of wide compatible varieties (WCVs) can be used in the development of intersubspecific hybrids and also for parental line development in hybrid rice breeding.

## 1. Introduction

The yield advantage of hybrid progenies over their parents, the heterosis, has contributed immensely to boosting the productivity of rice. In self-pollinated crops, heterosis is relatively weak compared to cross-pollinated crops, due to the dominion of homozygous balance [1]. Therefore, parental selection to promote cross-fertilization is of paramount importance in breeding hybrid rice. A significant breakthrough in hybrid rice breeding came with the discovery of wild-abortive (WA) cytoplasmic male sterility (CMS), which promoted a great deal of controlled cross-fertilization [2]. However, the WA system was found to work better within the *indica* subgroup than within the *japonica*, due to poor fertility restoration in the latter [3]. Using CMS systems, a significant effort has been placed into developing several rice hybrids in the last four decades worldwide. In India alone, 127 rice hybrids have been commercially released during the last three decades all in the *indica* background. Among the heterotic systems in rice, however, intersubspecific crosses are more heterotic than intrasubspecific crosses [4]. Yield heterosis in rice can be arranged in the descending order of sub-specific combinations as *indica*/temperate *japonica* > *indica*/tropical *japonica* > temperate *japonica*/tropical *japonica* > *indica*/*indica* > *japonica*/*japonica* [5]. The development of intersubspecific hybrids, particularly the *indica*/*japonica* combination, remains poorly resolved due to the major limitation of hybrid sterility. Although the singular or dual origin of rice is still debated [6,7], the subspecific differentiation is prominently associated with the reproductive barrier between *indica* and *japonica* subtypes [8]. Further divergence of phenotypes and adaptations among *indica* and *japonica* subspecies resulted in their genetic differentiation. Despite the heterotic potential, *indica*–*japonica* hybrids were unsuccessful due to several hindrances *viz.,* low seed setting, poor spikelet filling, and transgression of plant height and growth duration [9]. Therefore, it is important to break the crossability barrier between the two sub-species and harness the heterotic potential of the *indica*/*japonica* hybrids.

The major hindrance in producing viable hybrids from *indica*/*japonica* crosses is hybrid sterility, which occurs to varying degrees [10]. Sterility can occur either due to prezygotic or postzygotic mechanisms. The prezygotic mechanism prevents the formation of hybrid seed, while the postzygotic mechanisms occur after fertilization and seed formation and encompass hybrid necrosis, hybrid weakness, hybrid sterility/semi sterility and lethality. Hybrid sterility is one of the key forms of postzygotic reproductive isolation between *indica* and *japonica*, caused due to the failure of chromosome pairing at the meiosis stage abetted by the structural differences between parental chromosomes [11]. One of the major genetic systems regulating the reproductive isolation of *indica* and *japonica* is the triallelic system of the *S5* locus [12]. Popularly known as the wide compatibility (WC) system, the *S5* locus has three alleles, *S5-i*, *S5-j* and *S5-n* [13]. Localized to chromosome 6 are two specific alleles, *S5-i* to *indica* sub-species and *S5-j* to *japonica* sub-species. The third allele, the *S5-n* allele, is neutral and does not show any subspecies allegiance. Among the alleles, the hybridity of *S5-i* and *S5-j* alone leads to sterility, while all the other combinations result in fertile grains. Plants with the *S5-n*/*S5-j* (or *S5-i*) genotype are fully fertile, while plants with the *S5-i*/*S5-j* genotype have poor fertility. Specifically, the genotypes carrying *S5-n* alleles can cross with both *indica* and *japonica* lines effectively, and are therefore called wide compatible varieties (WCV). The *S5-n* allele has a 136 bp deletion in the *S5* gene, causing the deranged sub-cellular localization of the aberrant S5 protein and rendering the gene non-functional. Therefore, whenever a WCV (*S5-n*) was crossed with *indica* (*S5-i*) or *japonica* (*S5-j*) genotype, fertile hybrids would be produced [14]. Because of this ability, WCVs can act as the bridge for *indica*–*japonica* hybridization. WCVs have been known for a long time to the rice breeders as genotypes that cross freely with both *indica* and *japonica* lines. Therefore, WC has been recognised as one of the key requirements to overcome the fertility barrier among intersubspecific hybrids [15]. Moreover, WCVs can act as potential donors for *S5-n* alleles, which can replace either *S5-i* or *S5-j* alleles in the parental stocks to generate potentially viable hybrids. Integrating the portability of the *S5-n* allele into WA-CMS restorers that are locally well adapted and agronomically desirable can generate wide compatible restorer (WCR) lines. Conventionally, WCVs could be identified through test crossing and analysing the spikelet fertility of the F_1_ hybrids, which is tedious and time consuming. Moreover, identification on the basis of spikelet fertility data or by using other morphological traits associated with the WC gene may be inconclusive due to interactions from the external environment. The recent development of molecular markers linked to the *S5* locus could aid to overcome these limitations. A PCR-based co-dominant *S5* functional multiplex marker system (*S5*-MMS) could identify *indica* and *japonica* alleles based on the SNP they are carrying and also the *S5-n* allele based on the deletion [14].

Among the subspecies of *Oryza sativa*, there is a third group of genotypes that were characteristically distinct from the major subtypes. Known previously as *javanica* types, and later as tropical *japonica*, these groups of genotypes show an intermediary behaviour between *indica* and *japonica* [15]. Most of these types are also characteristically cross compatible with both *indica* and *japonica* genotypes and are known to harbour WC alleles. However, utilization of tropical *japonica* in hybrid rice breeding has been limited due to their lower productivity and other undesirable traits [16]. Further, the information on their natural diversity among these group of genotypes is restricted, and they are less well characterized at the molecular level [17]. Notwithstanding, the natural diversity and distribution of the *S5-n* allele are not limited to tropical *japonica*. Two earlier known WCVs, Dular and Nagina 22, come from the *aus* type of rice. To utilize the WC system in hybrid diversification in rice, it is necessary to use genotypes with suitable allele combinations. It may be difficult to utilize WCVs for developing hybrids directly, because of the undesirable traits they may carry. This requires pre-breeding to incorporate desirable alleles including WC into suitable backgrounds and then to improve parental lines for further hybrid breeding. Therefore, recruiting the *S5-n* allele into the parental stock should be considered a preliminary step for the development of heterotic *indica*/*japonica* hybrids. Moving towards this, in the present study, we have surveyed a diverse set of 950 rice genotypes, including tropical *japonica* lines and several other germplasm lines, for WC system diversity. Since not much information is available, other than for Dular and Nagina 22, we have further characterized the WCVs for their agronomic potential and hybrid fertility.

## 2. Results

### 2.1. Marker-Assisted Identification of Wide Compatible Varieties (WCVs)

**Figure 1:**
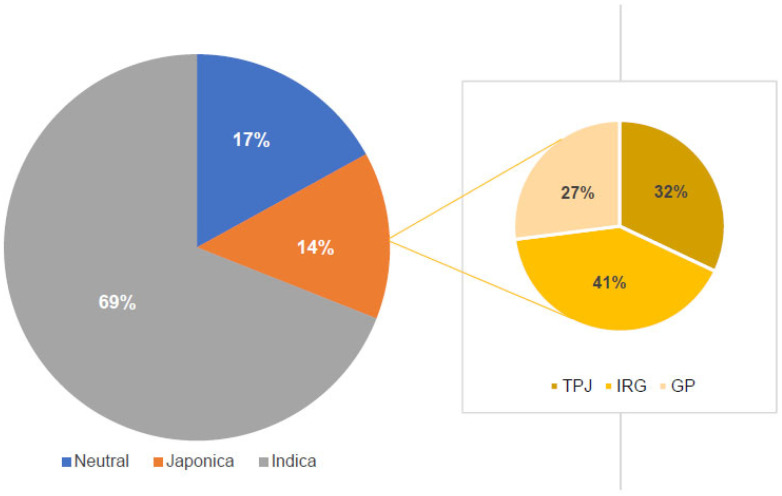
Frequency distribution of different alleles of *S5* locus *viz., S5-n*, *S5-i* and *S5-j* in the set of 950 rice genotypes. TPJ, tropical *japonica*; IRG, international rice germplasm; GP, local germplasm.

**Figure 2:**
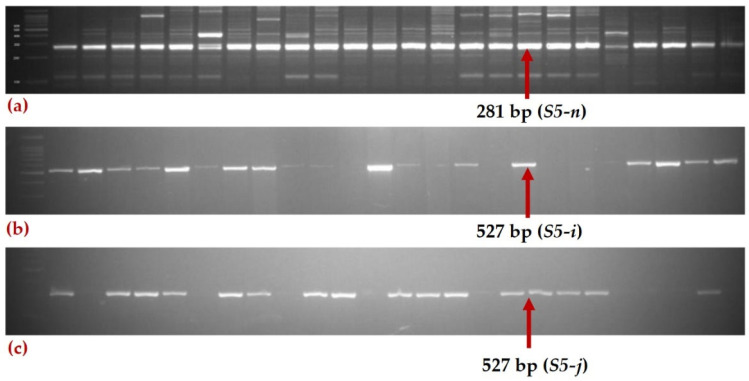
Representative gel images showing the amplification profile of functional markers for different alleles of *S5* locus *viz., S5-n*, *S5-i* and *S5-j* in the 967 lines. (**a**) Screening for *S5-n* allele using S5-InDel primer; (**b**) screening for *S5-i* allele using S5-ELSP; (**c**) screening for *S5-j* allele using S5-JASP1.

The 950 rice genotypes included 744 *indica* (78.3%), 188 *japonica* (19.7%), 14 Basmati (1.5%), and 4 *aus* types (0.4%) (Table 1). Out of 950 rice genotypes, 17% of the genotypes were identified as wide compatible varieties (WCVs) possessing a homozygous *S5-n* allele with a band size of 281 bp, 69% of the genotypes had the *S5-i* allele with a band size of 527 bp and 14% of the genotype were homozygous for the *S5-j* allele with a band size of 325 bp (Figure 1). Among these, 14% of *indica* genotypes possessed *S5-n* alleles, followed by 27% of *japonica* genotypes. Representative gel images for different *S5* alleles are presented in Figure 2. Among the 160 wide compatible varieties, 41% were IRG lines, 32% were *japonica* lines and the remaining 27% were rice germplasm (Table S4).

### 2.2. Variation in Morphological Traits among the Wide Compatible Varieties

Analysis of variance showed significant variation among the 92 WCVs for all twelve traits. A combined ANOVA was also calculated, which showed that the genotypic effect was significant for various traits *viz.,* plant height, panicle length, number of tillers per plant, filled grains per panicle, unfilled grains per panicle, total grains per panicle, spikelet fertility percentage, pollen fertility percentage, grain length, grain width, yield per plant and days to 50% flowering. However, the environmental effect was significant for plant height, panicle length, number of tillers per plant, filled grains per panicle, unfilled grains per panicle, and total grains per panicle. Significant GE interaction was observed for all the traits except panicle length (Table 2). The mean days to 50% flowering was recorded to be 90 days, with a minimum of 78 and a maximum of 116 days; the mean value for grain number was recorded as 175.8, with a range of 20.7 to 302.2; the spikelet fertility varied from 31.9% to 99.4%, with an average of 86.4%; the plant height of WCVs varied from 80 cm to 231.6 cm, with an average of 146.2 cm; and yield per plant varied from 4.1 gm per plant to 44.3 gm per plant, with an average of 18.6 gm per plant. The CV for all the traits ranged from 2–10%, except for unfilled grain with a CV of 17.6%, contributing the highest variation, followed by filled grains with a CV of 8.34% and the number of tillers with a CV of 8.02%. Plant height and grain length showed the lowest variation with a CV of 2.2%.

### 2.3. Relationship among Wide Compatible Varieties Based on Agro-Morphological Traits

**Figure 3:**
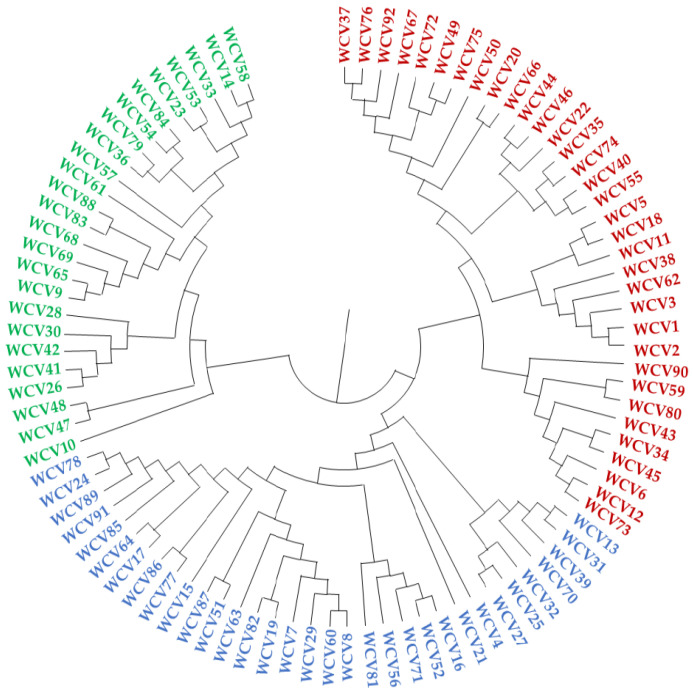
Dendrogram showing the morphological diversity among 92 WCVs based on morphological traits.

Based on the mean data on different morphological traits, the wide compatible varieties were grouped into three clusters (Figure 3). Among the clusters, cluster 3 had the highest number of lines (39 WCVs, 42.4%), followed by cluster 1 (33 WCVs, 35.9%) and cluster 2 (20 WCVs, 21.7%). The cluster centroids for different agro-morphological traits indicated that WCVs with more grain numbers and yield per plant were in cluster 2, followed by cluster 3 and cluster 1. Cluster 2 and cluster 1 possessed relatively taller plants than cluster 3, whereas WCVs with a greater number of tillers were found in cluster 1, followed by cluster 2 and cluster 3 (Table 3).

### 2.4. Genotypic Variation among the Wide Compatible Varieties

The 92 wide compatible lines along with four checks, namely P*S5*, PRR78, Pusa6B and Pusa44, were evaluated using 71 simple sequence repeat (SSR) markers distributed throughout the rice genome to determine the molecular diversity among the WCVs. Based on screening, 71 SSR markers generated a total of 199 different alleles with a minimum amplified product of 60 bp and a highest of 530 bp and the allelic range varied from 2 to 5 for a single locus. A total of 29 micro-satellite markers were bi-allelic, 29 markers were tri-allelic, 11 markers were tetra-allelic and 2 markers were penta-allelic. The highest numbers of alleles were observed in RM24 and RM152. Major allele frequency (MAF), which can be defined as alleles with more than 5% frequency, ranged from 0.3611 to 0.9063 with a mean MAF of 0.6133. RM144 had a minimum MAF of 0.3611 followed by RM6 (0.375), whereas qGN4.1 had a maximum MAF of 0.9063 followed by InDel-*S5* (0.8854) and RM26 (0.8646). The molecular genetic diversity index ranged from 0.1699 (qGN4) to 0.6963 (RM24); the average was 0.4909. The average heterozygosity was 0.00077, and ranged from 0 to 0.033. The polymorphic information content (PIC) ranged from 0.1555 to 0.647; the average was 0.41225. RM24 was found to be the most polymorphic marker in the genotype panel, with a PIC of 0.647, followed by RM144 (0.622), and RM19 (0.6205). Likewise, qGN4 was found to be least polymorphic with a PIC value of 0.1555, behind InDel-*S5* (0.1823). The overall information on genetic variability, PIC, and the total number of alleles is provided in Table 4.

### 2.5. Population Structure

**Figure 4:**
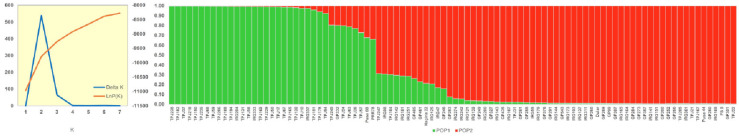
The maximum Δk estimate indicates the presence of two subpopulations among the wide compatible varieties.

The population structure of the WCVs indicated the presence of two distinct subpopulations (Figure 4). Within a group, wide compatible varieties with affiliation probabilities ≥50% were assigned to a distinct group and those with <95% were treated as ‘admixture’. A total of 71 WCVs had affiliation probabilities ≥95%, whereas 25 lines had shown mixed ancestry. A total of 35 genotypes (36.5%) were grouped into a first population (POP1), while 61 genotypes (63.5%) were grouped in the second population (POP2) (Figure 4).

Out of the total 25 admixed genotypes, 10 were from POP1 and 15 from POP2. The proportion of admixtures was more in POP1 (0.29) than in POP2 (0.25). The allele frequency divergence among the subpopulations was 0.134 (Table 5). The average expected heterozygosity between the individuals within POP1 was 0.47, while that of POP2 was 0.38. The fixation index (F*_ST_*) of POP1 was 0.15 and POP2 was 0.32. POP1 consisted of 7.4% of *indica* genotypes and 92.6% of *japonica* genotypes. Contrarily, POP2 accounted for 80.6% of *indica* lines and 19.4% of *japonica* lines along with two *aus* genotypes. Interestingly, 12 genotypes belonged to the phenotypic cluster 1 in POP1, which included 3 *indica* and 9 *japonica* lines. Likewise, 8 cluster 2 genotypes (all *japonica*), and 13 cluster 3 genotypes (1 *indica* and 12 *japonica*) were found in POP1. In POP2, there were 21 cluster 1 genotypes (18 *indica* and 3 *japonica*), 12 *indica* genotypes belonging to cluster 2 and 26 cluster 3 genotypes (20 indica, 4 *japonica*, and 2 aus). Among the checks used, Pusa 6B and PRR78 were grouped into POP1 and showed admixing with POP2 alleles. Among the aus-type WCVs, Nagina 22 showed apparent admixing, while Dular did not and belonged to POP2. In addition, the checks Pusa 44 and Pusa Sugandh 5 (P*S5*) showed no admixing and were grouped into POP2.

### 2.6. Graphical Clustering Based on Genetic Distance

**Figure 5:**
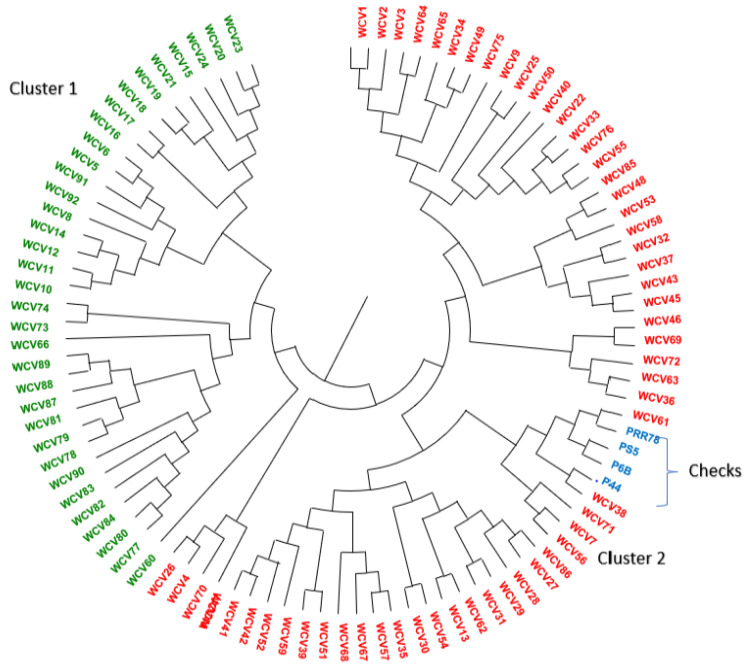
Representation of genetic distance among wide compatible varieties.

Based on the genetic distance estimated using simple matching coefficients, the WCVs fell into two major clusters (Figure 5), group 1 comprising 34 WCVs and group 2 comprising 58 WCVs including checks. Group 1 was predominantly made up of *japonica* genotypes (32) along with two *indica* varieties, Shah Pasand and Pusa 1176, whereas group 2 had an equal distribution of *indica* (30) and *japonica* (26) along with two *aus* varieties, Dular and Nagina 22.

### 2.7. Performance of Intersubspecific Hybrids across the Locations

A total of 164 hybrids were developed utilizing 41 wide compatible varieties with two *indica* and two *japonica* lines, namely Pusa44, IR64, TPJ29 and TPJ52. Analysis of variance showed that there was significant variation for genotypic effect for all the locations (Table 6). 

Further, hybrids were sub-grouped based on allelic status, namely *S5-nS5-I* and *S5-nS5-j*, and single-factor ANOVA was performed for spikelet fertility (%) between the groups. ANOVA revealed no significant variance between groups in each location. A similar analysis was also performed for spikelet fertility (%) based on the overall mean and a non-significant effect was observed between groups (Table S5). Based on overall mean performance, plant height of the inter sub-specific crosses derived from wide compatible varieties ranged between 85.0 cm (WCH141) and 146.00 cm (WCH113), with a mean value of 113.60. Crosses with IRGC15046 had a high mean value for plant height (117.5 cm) followed by IR64 (106.5 cm). As far as the number of tillers per plant is concerned, crosses derived from wide compatible varieties WCV86 (26) produced a greater number of tillers. Hybrids derived from IRGC137 (177.00 and 176.00), BJ-1 (171.6) and Lalan Kanda (166) were found to be superior for filled grain per panicle. Spikelet fertility percentage was higher in WCH15 (91.8), followed by WCH47 (91.2), WCH125 (90.9), WCH86 (90.4) and WCH16 (90.3). Hybrids developed using wide compatible varieties IRG137 (50.40 gm), IRG143 (48.8 gm), OYR128 (46.8 gm) and IRGC10658 (44.50 gm) were found to be promising across all three different locations (Table S6).

### 2.8. Multi-Locational Evaluation of Hybrids Using GGE Biplot

**Figure 6:**
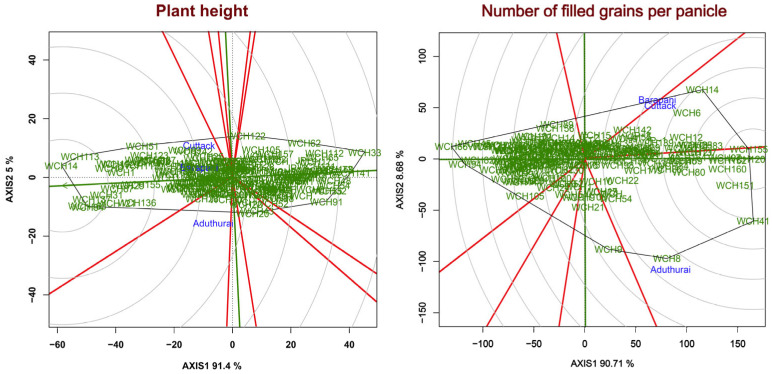
Combined biplots for multilocation performance wide compatible hybrids based on GGE biplot analysis on the most significant NPT related traits.

In the GGE biplot analysis, a stable genotype is placed closest to the average environment axis and is distributed over it based on the highest mean performance. The GGE biplot for most variable traits, namely plant height and filled grain in the present study, revealed that the majority of the variance is explained by the first two principal components; PC1 and PC2 explained 91.4% and 7.2% of variation, respectively. The genotypes WCH89, WCH51, and WCH113 (154.1 cm) had greater plant height at Cuttack, whereas WCH20 performed better at Aduthurai. The vector length of Barapani was less and it was unable to differentiate the genotypes for plant height. Based on ranking genotype WCH14, WCH113 had greater plant height and occupied the origin of the GGE graph (Figure 6). The total variance for filled grains was mostly explained by two principal components; 90.71% was explained by PC1 and 8.68% was explained by PC2. The genotypes WCH8 and WCH9 showed better performance for filled grains at Aduthurai, followed by WCH4, whereas WCH14 and WCH6 showed better performance at Barapani and Cuttack. Most of the genotypes showed stable performance across the environment and occupied origin. The ranking genotype graph showed that WCH155, WCH20 and WCH160 have more filled grain and occupy the origin of the GGE graph.

## 3. Discussion

Currently, the extent of rice cultivation in India is ~44 million hectares (mha), which is the highest in the world. With an annual production of ~118 million tonnes (mt), India ranks second after China in production. China produces 211.1 mt from 30.44 mha, a productivity of 69.3 q/ha as compared to India’s productivity of 23.9 q/ha [18]. The major reasons for China’s productivity can be attributed to the predominant cultivation of *japonica* rice, as well as to the formidable success in extensive cultivation of rice hybrids. India almost totally grows *indica* rice, which is less productive than *japonica*. Further, the extent of cultivation of hybrid rice is marginal (6.8%) in India, extending ~3.0 mha, as compared to ~48% in China [19]. The demand for rice in India is poised to take a quantum leap in the future, 16% by 2030, 41% by 2040, and 67% by 2050 from the current production level [20]. To bridge the ensuing gap in food demand, rice production needs to be boosted, and one of the probable approaches is extending hybrid rice cultivation. However, the popularity of rice hybrids in India has been very slow, primarily due to factors such as low marginal yield gain over pure line varieties, increased seed cost and the need for seed renewal at every cropping cycle [21]. To meet this challenge, heterosis in rice hybrids needs to be improved.

Rice hybrids are to be produced independently for different utilisation segments and ecologies to achieve sustained spread and extensive cultivation. Therefore, the diversification of parental lines for hybrid rice needs to be context specific. This needs a holistic approach with respect to the requirements of the target segment, including grain quality as well as general genetic systems that improve yield heterosis. If the indica–*japonica* hybrids can be realised with the same ease of producing three-line or two-line hybrids as currently practised in rice, the challenge of heterotic boosting can be met rather easily. Judicious deployment of the WC system is hence required to meet this task. However, the availability of WCVs across different rice segments is rather limited and comprehensive information is seldom available on the diversity of the WC genetic system. Further, only a limited number of WCVs have been utilized in the intersubspecific hybridization program [22]. This requires an extensive survey across various rice types and source WCVs within each group to develop/improve the parental stocks with WC. The availability of marker systems targeting the *S5-n* allele can simplify this task by integrating the allele through marker-assisted breeding.

More than 50 loci have been identified associated with hybrid sterility in rice [23]. Among all loci causing embryo sac abortion, the *S5* locus has been recognised as prominent in controlling indica–*japonica* hybrid compatibility. Therefore, the *S5* alleles pertaining to the two major subspecies ensure their reproductive isolation. However, the neutral allele, *S5-n* is found distributed in both *indica* and *japonica* types with varying frequencies. This study, despite being dominated by the *indica* genotypes, indicated an overall distribution of the *S5-n* allele with a frequency of 0.17. The allelic frequencies among the 950 genotypes tested were 0.69 for the *S5-I* allele and 0.14 for the *S5-j* allele. The *indica* genotypes possessed the *S5-n* alleles with a frequency of 14%, whereas the *japonica* genotypes indicated a larger proportion of *S5-n*, with a frequency of 27%. It may be pertinent here to emphasize that most of the *japonica* types used in this study came from the tropical *japonica* group, which is already known to harbour a relatively higher proportion of the *S5-n* allele [24]. Apart from this, the Basmati/aromatic group did not carry the WC allele. Almost all of the *indica* types contained *S5-i*, as expected, except for two IRG lines, IRG-17 and IRG-20. In the case of *japonica* types, *S5-j* alleles predominated. There were three *aus* genotypes that carried the *S5-n* allele. In an earlier study, Revathi et al. [24] found 46% of the genotypes possessing the *S5-n* allele using an S5-InDel marker, along with 0.06% of the heterozygotes. However, we could not find any *S5-n* heterozygotes in this evaluation. There are also reports of the presence of 19.9% WCVs among a germplasm set of 584 genotypes comprising 154 cultivars, 207 IRRI germplasm accessions, 37 aromatic genotypes, 157 restorer lines, 12 maintainer lines and 17 breeding lines [14]. All the genotypes that were identified to carry the *S5-n* alleles in this study can be construed as novel sources of WC, as they have never been intentionally used for intersubspecific hybridisation. These 150 WCVs can further be utilized in parental diversification and the development of indica/*japonica*-based breeding programmes.

For further utilization of the WCVs, it is essential to characterise them agronomically and genetically. Genetic diversity could help classify the genotypes into different target classes, either agronomically, such as duration, yield and plant stature, or genetically, into subpopulations. In order to do this, we further pooled the 160 WCVs identified to draw a random subset for diversity analysis. The subset contained 92 WCVs along with four checks for agronomic and molecular evaluation. Agronomically, the genotypes displayed a three-cluster formation. Irrespective of the genetic grouping based on genomic differences, the agronomy-based grouping helped us to practically utilise the genotypes for breeding based on the similarity/contrast of quantitative and morphological traits [25]. This aids in formulating crossing schemes considering flowering synchrony, as well as by matching the duration classes. High- and low-yielding types can also be distinguished. Moreover, photo-sensitive and -insensitive lines can be distinguished for choosing the breeding seasons. Therefore, morphology-based grouping can supplement the genetic grouping in designing effective breeding strategies for forming an intersubspecific breeding program [26]. Using a similar strategy, Kumar et al. [27] grouped iso-cytoplasmic rice restorers derived from the commercial hybrids into two major groups based on morphological traits. The number of groups based on agronomic traits often varies from the genetic groups because of the extreme phenotypic variation that can occur within a genetically similar class. There are earlier reports in which a small group of 41 rice genotypes were grouped into 6 clusters based on 13 morphological traits [28].

As emphasised above, molecular marker-based diversity studies provide the opportunity to classify the population based on genomic similarities. Genotypes based on 71 SSR markers and 92 WCVs fell into two subpopulations in both the graphical and model-based approaches. The estimated genetic diversity of the markers indicated sufficient resolving power suitable for genetic groping of the WCVs. The overall genetic distance revealed that the genetic variability present among these lines was moderate, with 2.80 alleles per marker and an average PIC value of 0.41, with an overall Nei’s gene diversity of 0.49. The average allele number per marker found among the WCVs was similar to that recorded from genotype sets with a narrow genetic base, such as Japanese [29] and Korean cultivars [30], but lower than from diverse germplasm sets [31,32,33,34,35]. The allele frequency divergence between the subpopulations or subgroups of genotypes indicates the population structure [36,37]. The genotypes from different subpopulations are assumed to be descended from different sets of ancestors that have become isolated in the evolutionary process. Therefore, there may be common alleles distributed across the whole population along with specific alleles that are characteristic of subpopulations. Therefore, an admixed model with correlated allelic frequencies is preferred in estimating the population structure. The allelic frequencies either use identical by descent or identical by state probabilities. The assumed subpopulations (K) therefore show a maximum difference in the log probability at each K, Pr(X|K) at the best K [38]. Accordingly, the two subpopulations identified in this study, one with 31 genotypes and the other with 49 genotypes indicated a large level of admixing between the subpopulations. The same Bayesian model-based approach has been used to study the population structure of plant populations by various researchers. In most of the studies on rice subpopulations, two subpopulations are most commonly detected, pertinently due to indica–*japonica* subspecific differentiation [39]. However, there are reports of up to 7 subpopulations reported among 416 rice accessions collected from China [33]. Besides, the admixtures are common in rice subpopulations, indicating high admixing between populations. However, there are currently no studies reporting the population structure of WCVs, except for the inclusion of a few genotypes among the large populations studied [40]. Genetic differentiation among WCV subpopulations indicated that they are moderately divergent and contained admixtures (28.6% in POP1 and 24.6% in POP2). Based on genetic differentiation among the wide compatible varieties identified, within-group variability was high among the subpopulations. The genetic diversity identified herein may not reflect the phenotypic diversity effectively, since the SSR markers are mainly present in non-coding regions [41]. The use of random markers for assessing genetic diversity might not reflect the functionally useful variations prevalent in the coding regions of the genome [42], and thus may not classify genotypes based on the quantitative expression of traits. Nevertheless, the overall genetic diversity would be useful in selecting genetically diverse parents for breeding programmes. Phenotypic analysis, therefore, needs to be integrated into genetic variations for ensuring the effective use of functional variants in breeding. The selection of parental lines from distant clusters would help in improved heterosis, which may further improve the yielding potential of hybrids.

The agronomic performance of WCVs indicated that they can be grown in wider ecologies, which is essential for their effective utilisation as WC donors. Donor genotypes with lesser diversity with the target set of recipients can help in improved breeding efficiency due to fewer alterations in the target traits and leverages the recovery of the recipient genome.

Yet, WCVs are to be evaluated for their ability in cross compatibility. We have selected a subset of 41 genotypes to test this, and the selection was made based on their flowering behaviour and plant type. Two *indica* parents, Pusa 44 and IR64, were selected along with two *japonica* parents, IRGC8146 and IRGC15046, and the 164 hybrids showed average spikelet fertility of 70.5%, implying that the WCVs identified in the study are effective and useable in breeding. Hybrids also showed significant variation for other agronomic traits, as well as varying stability levels. Hybrids and checks showed differential behaviour with changes in the environment. Therefore, the hybrids were evaluated across three locations and it was found that the majority of the hybrids had excellent spikelet fertility (%), with an average of 70.53%. Dular and Nagina 22 have been extensively utilized in inter sub-specific hybridization by Vijayakumar et al. [22]. Here, we also aimed to identify superior wide compatible varieties with higher spikelet fertility than the existing ones. We have identified a few combinations, namely WCH 41, WCH 73, WCH 120, WCH 36 and WCH 80, which showed higher spikelet fertility (%) than hybrids derived from Dular and Nagina 22. Our results justify the selection of parental lines for the development of intersubspecific hybrids. The average mean value was higher for spikelet fertility when *indica* testers were crossed with *indica* lines carrying neutral alleles, whereas in the case of the *japonica* tester, higher spikelet fertility was observed when crossed with *japonica* lines carrying the neutral allele. Additionally, average spikelet fertility was higher (72.3%) when *japonica* lines were used as the female parent. In our study, we used all wide compatible varieties as male parents and found higher expression of spikelet fertility. Vijayakumar et al. [22] observed higher expression of traits in hybrids when the WC gene in a male parent was used.

To study the variation in spikelet fertility between *S5-i*/*S5-n* and *S5-j*/*S5-n*, F_1_ hybrids were classified into two groups. In this study, 82 hybrids were derived from crosses between 41 wide compatible varieties with two *indica* lines, and another 82 hybrids were derived from crosses between 41 wide compatible varieties with two *japonica* lines, and it was observed that there were no significant differences between the groups with respect to spikelet fertility (%). Therefore, the potentiality of identified WCVs is assumed and the utility of a PCR-based functional marker (InDel *S5*) is shown for quick and precise identification of wide compatible varieties. These identified superior wide compatible varieties can further be used as a novel source of WC genes and will be utilized for marker-assisted incorporation of neutral alleles into elite rice varieties and the development of inter sub-specific hybrids.

## 4. Materials and Methods

A total of 950 genotypes, which included 178 tropical *japonica* lines, 293 IRG (International Rice Germplasm) lines collected from IRRI, along with 479 germplasm lines (released varieties) maintained at the Division of Genetics, ICAR-IARI, were used for the study. The details of the lines used are provided in Table S1.

### 4.1. Molecular Screening of Germplasm for Wide Compatibility Gene (S5-n)

Initially, all the 950 genotypes were subjected to screening using PCR-based functional markers, namely *S5-*InDel for the neutral allele, *S5*-ELSP for the *indica* allele and S5-JASP1 for the *japonica* allele, to analyse the allelic status at the *S5* locus. The list of primers used for screening germplasm is provided in Table S2. Three-week-old seedlings were used to isolate genomic DNA using a standard protocol [43]. A 10 µL reaction volume was prepared to perform polymerase chain reaction (PCR). Initially, template DNA was denatured at 94 °C (5 min), and then continuous PCR amplification (35 cycles) was performed. These 35 cycles of PCR amplification consist of three steps, denaturation at 94 °C for 30 s, annealing for 1 min at 55 °C and primer extension for 1 min at 72 °C, with a final extension for 7 min at 72 °C; the product was then cooled to 4 °C. The amplicons were analysed through electrophoresis in a 3.5% agarose gel mixed with 0.1 mg/mL using TAE buffer (1×). Based on the banding pattern of the gel, the lines were scored as indica, *japonica*, neutral or heterozygous types.

### 4.2. Phenotyping

The 92 wide compatible genotypes identified along with Dular and Nagina 22 were grown in two replications in a randomized block design at three different locations: IARI-ICAR, Experimental farm, New Delhi during *Kharif* 2020; off-season 2020 at RBGRC, ICAR-IARI Research station, Aduthurai, Tamil Nadu; and ICAR-IARI Research station, Karnal, Haryana during *Kharif* 2020. Phenotypic data were recorded on five healthy plants for each of wide compatible genotypes for twelve agro-morphological traits, namely days to 50% flowering, plant height, panicle length, number of tillers, number of filled grains per panicle, number of unfilled grains per panicle, the total number of grains per panicle, spikelet fertility (%), pollen fertility (%), grain length, grain width and yield per plant. Agro-morphological traits were subjected to analysis of variance (ANOVA). All the analyses were carried out using the R statistical environment [44].

### 4.3. Genotyping

A total of 71 microsatellite markers (Table S3), which are evenly distributed across 12 chromosomes for assessing molecular diversity, were used for the estimation of genetic relation among 92 wide compatible genotypes along with 4 checks, namely P*S5*, PRR78, Pusa6B and Pusa44.

### 4.4. Diversity Analysis

The statistical analysis consists of, alleles/locus, major allele frequency, expected and observed heterozygosity, gene diversity and polymorphic information content (PIC). The molecular distance-based clustering was performed with UPGMA by using Power Marker v3.25 [45]. Polymorphic information content was calculated as per the formula given by [46] for self-pollinated crops and further modified by [47].

$${\mathrm{PIC}}_{i}=1-\overset{n}{\underset{j=1}{\sum }}P_{\mathrm{ij}}^{2}$$

where P_ij_ is the proportion of the jth allele of the ith marker.

Genetic distance between the tropical *japonica* accessions was computed using Rogers distance [48] and the distance matrix was subjected to hierarchical clustering using the unweighted pair group method using averages (UPGMA). The dendrogram was constructed using Power Marker v3.25 and visualized using MEGA X.

### 4.5. Determination of Population Structure

It is established that different gene frequencies among the individuals in a population can stratify them into several subgroups. These differences may arise due to geographical isolation or any sort of selection performed on a population i.e., artificial or natural. To determine the geographical effects on the population structure of tropical *japonica* lines, pairwise F*_ST_* values were computed between each geographical group. Analysis was performed using PopGene Version 1.32 [49]. The pairwise F*_ST_* values were used for Euclidean clustering using hierarchical agglomerative methods, such as UPGMA, and visualized using the R statistical environment. Further, to detect the cumulative population structure of the test panel, Bayesian modelling of genotype data was used. The analysis assumes an admixture model for the population with correlated allelic frequencies. Analysis was carried out using Structure v.2.3.4. To detect the number of subpopulations, the structure generates a matrix of co-ancestry coefficients (Q matrix) that explains the probabilities of an individual falling into a subpopulation. The optimum number of subpopulations was determined using simulation summary statistics, by determining the ΔK using an ad hoc procedure [50]. Analysis was performed using the online tool Structure Harvester [51].

### 4.6. Development and Evaluation of Inter Sub-Species Hybrids across Three Locations

A total of 164 inter sub-specific hybrids were developed utilizing 41 wide compatible varieties by hybridizing with two *indica* parents, Pusa44 and IR64, and two *japonica* parents, TPJ29 (IRGC8146) and TPJ52 (IRGC15046). These hybrids were grown for multi-locational evaluation at three locations: Aduthurai, Cuttack and Barapani. Morphological data were recorded on five healthy plants for each wide compatible hybrid for seven agro-morphological traits: days to plant height, panicle length, number of tillers, number of filled grains per panicle, number of unfilled grains per panicle, spikelet fertility (%) and yield per plant. We tested the hybrids for their stability in performance across the multiple locations using the GGE biplot approach [52].

## 5. Conclusions

The functional marker system was found to be highly effective in the screening of rice germplasms to identify wide compatible varieties that will greatly reduce labour costs and time. A novel panel of WCVs identified in the study could be used to incorporate the WC gene into elite genotypes, including elite maintainer lines (B line) with either an *indica* or *japonica* background. Based on the diversity analysis, the diverse lines belonging to the different clusters can be evaluated and used as parents for developing superior intersubspecific hybrids. The wide compatible varieties identified in the present study would be useful in obtaining the fertile hybrids between *indica* and *japonica* crosses and in combining the superior traits of both the sub-species.

## Figures and Tables

**Table 1:** Distribution of *S5* alleles in the germplasm assembly.

Class	Number	Allelic Frequency		
		*S5-i*	*S5-j*	*S5-n*
*indica*	744	637	2	105
*Japonica*	188	0	137	51
Basmati/aromaitc	14	13	1	0
*Aus*	4	1	0	3

**Table 2:** Pooled ANOVA for twelve different agro-morphological traits.

Source	DFF	PH	PL	NT	FG	UFG	TG	SF (%)	PF (%)	YPP	GL	GW
Location (L)	94.6	8519.1 *	206.1 *	189.1 *	14585.4 *	265.0 *	18781.2 *	33.5 *	1005.5	422.8 *	0.3	0.1
WCVs (V)	238.5 *	4216.9 *	62.8 *	90.2 *	10131.4 *	1715.8 *	14820.0 *	261.9 *	221.8 *	327.1 *	10.0 *	0.8 *
L × V	9.8 *	26.3 *	1.8	1.8 *	316.6 *	52.1 *	406.8 *	14.9 *	13.2 *	9.7 *	0.3 *	0.0 *
Mean	90.1	146.2	23.6	9.5	148.9	26.9	175.8	85.1	86.4	18.6	8.7	2.5
Min	78.0	80.0	10.6	3.0	20.7	2.2	56.0	31.9	58.9	4.1	5.1	1.3
Max	116.0	231.6	32.9	24.4	302.2	132.9	344.1	98.3	99.4	44.3	12.9	3.4
CV%	2.4	2.2	6.47	8.02	8.34	17.6	7.76	3.6	3.3	7.6	2.2	6.0

DFF, days to 50% flowering; PH, plant height in cm; PL, panicle length in cm; NT, number of tillers per plant; FG, number of filled grains; UFG, number of unfilled grains; TG, total number of grains; SF, spikelet fertility in %; PF, pollen fertility in %; YPP, yield per plant in g; GL, grain length in mm; GW, grain width in mm; * significant variance at 5% level.

**Table 3:** Cluster centroids for different agro-morphological traits.

Cluster	DFF	PH	PL	NT	FG	UFG	TG	SF (%)	PF (%)	YPP	GL	GW
1	88.31	147.76	23.32	10.70	111.23	18.53	129.77	85.77	87.30	18.15	8.98	2.54
2	91.40	149.57	25.11	8.93	210.16	37.42	247.58	85.22	87.02	21.84	8.81	2.43
3	90.42	143.01	23.11	9.00	146.90	28.85	175.75	84.02	86.07	17.56	8.49	2.53
SE	0.67	2.78	0.34	0.41	4.31	1.76	5.17	0.71	0.63	0.77	0.14	0.04

DFF, days to 50% flowering; PH, plant height in cm; PL, panicle length in cm; NT, number of tillers per plant; FG, number of filled grains; UFG, number of unfilled grains; TG, total number of grains; SF, spikelet fertility in %; PF, pollen fertility in %; YPP, yield per plant in g; GL, grain length in mm; GW, grain width in mm.

**Table 4:** Chromosome-wise summary of genetic variation based on 71 microsatellite markers.

Chromosome	Markers	Allele Number		MAF	GD	PIC
		Range	Mean			
1	10	2–5	3.10	0.57	0.54	0.46
2	9	2–3	2.56	0.56	0.53	0.44
3	11	2–3	2.73	0.67	0.44	0.36
4	1	2	2.00	0.91	0.17	0.16
5	9	2–4	2.78	0.67	0.46	0.39
6	5	2–3	2.20	0.67	0.42	0.34
7	3	2–3	2.67	0.63	0.46	0.39
8	8	2–5	3.00	0.60	0.51	0.44
10	5	2–4	2.80	0.59	0.50	0.40
11	5	2–4	2.80	0.53	0.55	0.47
12	2	4	4.00	0.57	0.56	0.50

MAF, major allele frequency; GD, genetic distance; PIC, polymorphism information content.

**Table 5:** Subpopulation genetic parameters and distribution of various classes of WCVs and agronomic clusters among the subpopulations.

Particulars	POP1			POP2		
	CI	CII	CIII	CI	CII	CIII
*indica*	3	0	1	18	12	20
*japonica*	9	8	12	3	0	4
*Aus*	0	0	0	0	0	2
Total members	12	8	13	21	12	26
Membership proportion	0.359			0.641		
Proportion of admixtures	0.242			0.254		
Distance within cluster (H*e*)	0.466			0.38		
F*_ST_*	0.148			0.327		
Distance between cluster	0.134					

H*e*, expected heterozygosity; F*_ST_*, fixation statistic.

**Table 6:** Combined ANOVA for different agro-morphological traits based on evaluation of 164 hybrids along with 4 checks in augmented block design across 3 environments.

Trait	WC/*indica*		WC/*japonica*		Variance		
	Mean	Range	Mean	Range	Geno	Loc	GxL
Tiller number	9.4	4.3–24.1	10.0	4.0–25.2	7.9 **	10.82 **	9.16 **
Plant height	115.4	91.7–151.9	121.0	94.7–148.0	47.1 **	55.40 **	86.2 **
Panicle length	23.3	17.1–30.8	22.8	18.3–29.7	5.8 **	4.90 **	9.13 **
Filled grain	93.8	21.3–192.2	99.0	21.1–189.8	132.9 **	66.30 **	458.1 **
Unfilled grain	37.5	6.1–122.3	40.8	9.0–152.5	64.5 **	59.90 **	296.6 **
Spikelet fertility	71.8	22.2–91.5	72.3	17.6–90.0	13.5 **	6.6 **	104.3 **
Yield per plant	21.5	4.7–47.1	22.1	6.7–53.5	5.8 **	3.0 **	3.8 **

** *p* ≤ 0.00; Geno, genotype; Loc, location; WC, wide compatible.

## Data Availability

The data presented in this study are available in the Supplementary Materials.

## Supplementary Materials

The following supporting information can be downloaded at: https://www.mdpi.com/article/10.3390/plants11151930/s1, Table S1: Genotyping of 950 different rice lines for *S5* locus based on three functional markers *viz.,* S5-InDel, S5-IASP2 and S5-JASP1; Table S2: List of primers used for screening of the rice germplasm; Table S3: List of 71 standard SSR primers used for genetic diversity analysis; Table S4: List of wide compatible varieties identified; Table S5: Mean of hybrids for spikelet fertility (%) in *indica* and *japonica* subgroups; Table S6: Mean performance of hybrids across three locations.
