# Supplementary material for: Population Dynamics of Wide Compatibility System and Evaluation of Intersubspecific Hybrids by indica-japonica Hybridization in Rice
Source: Plants (Basel). 2022 Jul 26;11(15):1930. doi: 10.3390/plants11151930 (PMC9332614; doi:10.3390/plants11151930)
Supplement: Supplementary file 1 [file plants-11-01930-s001.zip › plants-1643545-supplementary.pdf]

Table S1: Genotyping of 950 different rice lines for *S5* locus based on three functional markers *viz*., S5-InDel, IASP2 and JASP1

Genotype	Allelic status	Genotype	Allelic status	Genotype	Allelic status	Genotype	Allelic status	Genotype	Allelic status
IRGC289	*j*	IRGC15092	*j*	IRGC69845	*j*	IRGC53087	*j*	IRG-21	*n*
IRGC328	*j*	IRGC15100	*j*	IRGC69708	*j*	IRGC51498	*n*	IRG-22	*n*
IRGC7486	*n*	IRGC16073	*j*	IRGC69367	*n*	IRGC23754	*j*	IRG-23	*i*
IRGC7756	*j*	IRGC10658	*n*	IRGC67846	*j*	TPJ 201	*n*	IRG-25	*i*
IRGC8193	*j*	IRGC8182	*n*	IRGC67437	*j*	TPJ 203	*n*	IRG-26	*i*
IRGC3223	*j*	IRGC5726	*n*	IRGC66745	*j*	TPJ 204	*j*	IRG-27	*i*
IRGC15147	*j*	IRGC5766	*j*	IRGC26872	*j*	TPJ 205	*j*	IRG-28	*i*
IRGC1797	*n*	IRGC137	*n*	IRGC26178	*j*	TPJ 206	*n*	IRG-31	*i*
IRGC5320	*n*	IRGC1220	*j*	IRGC25966	*j*	TPJ 207	*j*	IRG-33	*i*
IRGC4059	*j*	IRGC1723	*j*	IRGC25759	*j*	TPJ 209	*j*	IRG-34	*i*
IRGC3849	*n*	IRGC6937	*n*	IRGC25239	*j*	TPJ 210	*j*	IRG-35	*i*
IRGC3764	*j*	IRGC1972	*n*	IRGC25510	*j*	TPJ 211	*j*	IRG-36	*i*
IRGC3408	*j*	IRGC1739	*n*	IRGC25660	*n*	TPJ 214	*n*	IRG-37	*i*
IRGC3394	*j*	IRGC1819	*j*	IRGC66758	*n*	TPJ 216	*j*	IRG-38	*i*
IRGC3370	*j*	IRGC1789	*j*	IRGC67428	*j*	TPJ 217	*j*	IRG-39	*i*
IRGC2263	*j*	IRGC1790	*j*	IRGC61841	*j*	TPJ 218	*j*	IRG-40	*n*
IRGC5769	*j*	IRGC16081	*j*	IRGC57692	*j*	TPJ 221	*j*	IRG-41	*i*
IRGC10365	*j*	IRGC17052	*j*	IRGC62162	*j*	TPJ 223	*j*	IRG-42	*i*
IRGC6309	*j*	IRGC17051	*j*	IRGC63265	*j*	TPJ 224	*j*	IRG-43	*i*
IRGC6457	*n*	IRGC16449	*j*	IRGC63121	*j*	TPJ 226	*n*	IRG-44	*i*
IRGC5441	*n*	IRGC17906	*n*	IRGC57781	*j*	TPJ 227	*n*	IRG-46	*i*
IRGC5097	*n*	IRGC19462	*j*	IRGC66760	*j*	TPJ 228	*n*	IRG-47	*n*
IRGC4122	*n*	IRGC18936	*n*	IRGC66756	*j*	TPJ 229	*j*	IRG-49	*i*
IRGC8196	*j*	IRGC18425	*j*	IRGC66644	*j*	TPJ 232	*j*	IRG-50	*i*
IRGC8261	*n*	IRGC1822	*j*	IRGC66630	*j*	TPJ 233	*n*	IRG-51	*i*
IRGC8269	*j*	IRGC19919	*j*	IRGC66529	*n*	TPJ 235	*j*	IRG-53	*i*
IRGC8146	*j*	IRGC23364	*j*	IRGC50399	*n*	TPJ 237	*j*	IRG-54	*i*
IRGC2245	*j*	IRGC24275	*j*	IRGC48733	*j*	TPJ 238	*j*	IRG-55	*n*
IRGC10758	*n*	IRGC18021	*j*	IRGC48493	*j*	TPJ 241	*n*	IRG-56	*n*
IRGC2455	*j*	IRGC22712	*j*	IRGC47345	*j*	TPJ 242	*n*	IRG-58	*i*
IRGC10760	*j*	IRGC8266	*j*	IRGC43394	*j*	TPJ 244	*j*	IRG-59	*i*
IRGC11010	*j*	IRGC65646	*j*	IRGC43372	*j*	TPJ 245	*j*	IRG-60	*i*
IRGC11169	*j*	IRGC64914	*j*	IRGC40199	*j*	TPJ 247	*n*	IRG-61	*n*
IRGC11336	*n*	IRGC64911	*n*	IRGC38994	*n*	TPJ 248	*j*	IRG-62	*n*
IRGC11821	*j*	IRGC64896	*j*	IRGC35724	*j*	TPJ 249	*i*	IRG-63	*i*
IRGC12052	*j*	IRGC64888	*j*	IRGC34018	*j*	TPJ 250	*i*	IRG-64	*n*
IRGC14530	*j*	IRGC64858	*j*	IRGC32411	*j*	TPJ 192	*i*	IRG-65	*n*
IRGC14694	*j*	IRGC64763	*j*	IRGC32406	*n*	IRG-1	*i*	IRG-66	*i*
IRGC14725	*j*	IRGC64657	*j*	IRGC32388	*n*	IRG-2	*i*	IRG-67	*n*
IRGC14738	*n*	IRGC64656	*j*	IRGC31051	*n*	IRG-9	*i*	IRG-68	*i*
IRGC14779	*j*	IRGC71559	*n*	IRGC30921	*n*	IRG-11	*n*	IRG-69	*i*
IRGC14791	*j*	IRGC71544	*j*	IRGC27321	*j*	IRG-12	*i*	IRG-70	*i*
IRGC14917	*j*	IRGC71493	*j*	IRGC24528	*n*	IRG-14	*i*	IRG-71	*i*
IRGC15006	*n*	IRGC69911	*n*	IRGC6741	*n*	IRG-15	*i*	IRG-73	*i*
IRGC15023	*j*	IRGC69910	*j*	IRGC24274	*n*	IRG-17	*j*	IRG-74	*i*
IRGC15046	*j*	IRGC69861	*j*	IRGC55808	*n*	IRG-18	*n*	IRG-75	*n*
IRGC15073	*j*	IRGC69857	*j*	IRGC54201	*j*	IRG-20	*j*	IRG-76	*i*

Table S1 *(Contd…)*

Genotype	Allelic status	Genotype	Allelic status	Genotype	Allelic status	Genotype	Allelic status	Genotype	Allelic status
IRG-77	*i*	IRG-128	*n*	IRG-183	*i*	IRG-265	*i*	IRG-318	*i*
IRG-78	*i*	IRG-129	*i*	IRG-185	*n*	IRG-267	*i*	IRG-320	*i*
IRG-79	*n*	IRG-130	*i*	IRG-187	*i*	IRG-268	*i*	IRG-322	*i*
IRG-80	*i*	IRG-131	*i*	IRG-190	*i*	IRG-269	*i*	IRG-323	*i*
IRG-81	*i*	IRG-133	*n*	IRG-193	*i*	IRG-270	*i*	IRG-324	*n*
IRG-82	*n*	IRG-134	*i*	IRG-196	*i*	IRG-271	*n*	IRG-325	*i*
IRG-83	*i*	IRG-135	*i*	IRG-198	*i*	IRG-272	*i*	IRG-326	*n*
IRG-84	*i*	IRG-136	*i*	IRG-200	*n*	IRG-273	*i*	IRG-327	*i*
IRG-85	*i*	IRG-137	*n*	IRG-201	*i*	IRG-274	*i*	IRG-328	*i*
IRG-86	*i*	IRG-138	*i*	IRG-204	*n*	IRG-275	*i*	IRG-329	*i*
IRG-87	*i*	IRG-139	*n*	IRG-205	*i*	IRG-276	*i*	IRG-333	*n*
IRG-88	*i*	IRG-140	*i*	IRG-211	*i*	IRG-277	*i*	IRG-335	*i*
IRG-89	*i*	IRG-141	*n*	IRG-212	*i*	IRG-278	*i*	IRG-337	*n*
IRG-90	*i*	IRG-142	*n*	IRG-214	*i*	IRG-280	*i*	IRG-338	*i*
IRG-91	*i*	IRG-143	*n*	IRG-215	*i*	IRG-281	*i*	IRG-340	*i*
IRG-92	*n*	IRG-144	*n*	IRG-217	*i*	IRG-282	*i*	IRG-341	*n*
IRG-93	*i*	IRG-149	*i*	IRG-218	*i*	IRG-283	*i*	IRG-342	*i*
IRG-94	*i*	IRG-150	*n*	IRG-219	*n*	IRG-284	*i*	IRG-343	*i*
IRG-95	*i*	IRG-151	*i*	IRG-223	*i*	IRG-285	*i*	IRG-345	*i*
IRG-96	*i*	IRG-152	*i*	IRG-224	*i*	IRG-286	*i*	IRG-346	*i*
IRG-97	*i*	IRG-153	*i*	IRG-227	*i*	IRG-287	*i*	IRG-347	*i*
IRG-98	*n*	IRG-154	*i*	IRG-229	*i*	IRG-288	*i*	IRG-348	*i*
IRG-99	*i*	IRG-155	*i*	IRG-230	*i*	IRG-289	*i*	IRG-351	*i*
IRG-101	*i*	IRG-156	*i*	IRG-231	*i*	IRG-290	*i*	IRG-353	*n*
IRG-102	*i*	IRG-157	*i*	IRG-232	*i*	IRG-291	*i*	IRG-354	*i*
IRG-103	*i*	IRG-158	*i*	IRG-233	*i*	IRG-292	*i*	IRG-356	*i*
IRG-104	*i*	IRG-159	*i*	IRG-237	*i*	IRG-293	*i*	IRG-361	*n*
IRG-105	*i*	IRG-160	*i*	IRG-239	*n*	IRG-294	*i*	IRG-363	*i*
IRG-106	*i*	IRG-161	*i*	IRG-240	*i*	IRG-295	*i*	IRG-364	*i*
IRG-107	*n*	IRG-162	*i*	IRG-241	*i*	IRG-296	*i*	IRG-365	*i*
IRG-108	*i*	IRG-163	*i*	IRG-242	*n*	IRG-297	*i*	IRG-366	*i*
IRG-109	*i*	IRG-164	*n*	IRG-243	*n*	IRG-298	*n*	IRG-368	*i*
IRG-110	*n*	IRG-165	*n*	IRG-244	*n*	IRG-299	*i*	IRG-369	*i*
IRG-112	*n*	IRG-166	*i*	IRG-245	*n*	IRG-300	*i*	IRG-371	*i*
IRG-113	*i*	IRG-167	*n*	IRG-247	*n*	IRG-303	*i*	IRG-373	*i*
IRG-114	*i*	IRG-168	*i*	IRG-248	*i*	IRG-304	*i*	IRG-374	*n*
IRG-115	*n*	IRG-169	*n*	IRG-249	*n*	IRG-305	*i*	IRG-377	*i*
IRG-116	*n*	IRG-170	*i*	IRG-251	*n*	IRG-306	*i*	IRG-379	*i*
IRG-117	*i*	IRG-171	*i*	IRG-254	*i*	IRG-307	*i*	IRG-381	*i*
IRG-118	*i*	IRG-173	*n*	IRG-255	*n*	IRG-308	*i*	IRG-383	*i*
IRG-120	*i*	IRG-174	*i*	IRG-256	*i*	IRG-309	*i*	IRG-384	*i*
IRG-121	*n*	IRG-175	*i*	IRG-257	*i*	IRG-311	*n*	IRG-385	*i*
IRG-122	*i*	IRG-176	*i*	IRG-258	*n*	IRG-312	*i*	IRG-387	*i*
IRG-123	*i*	IRG-177	*i*	IRG-259	*i*	IRG-313	*i*	IRG-388	*i*
IRG-124	*i*	IRG-178	*i*	IRG-260	*i*	IRG-314	*i*	Sneha	*i*
IRG-125	*n*	IRG-179	*n*	IRG-262	*i*	IRG-315	*i*	Heera	*i*
IRG-126	*i*	IRG-180	*i*	IRG-263	*i*	IRG-316	*i*	IRBB 1	*i*
IRG-127	*n*	IRG-181	*n*	IRG-264	*i*	IRG-317	*i*	Rudra	*i*

Table S1 *(Contd…)*

Genotype	Allelic status	Genotype	Allelic status	Genotype	Allelic status	Genotype	Allelic status
Nipponbare	*j*	FL 478	*i*	IRBB 14	*i*	Manhar	*i*
Lalitagiri	*i*	Pant Dhan 11	*i*	IRBB 8	*i*	Vikash	*i*
C101PKT	*i*	Himalaya2	*i*	PR 106	*i*	Sidhanta	*i*
Jaldi Dhan 6	*i*	Kalinga-I	*i*	IRBB 60	*i*	Prasad	*i*
Jaldi Dhan 13	*i*	K 332	*i*	IRBB-59	*i*	IR-64	*i*
Shankar	*i*	Rasi	*i*	IRBB 13	*i*	Pant Sankar Dhan	*i*
Ghanteshwari	*i*	ADT 45	*i*	IRBB 55	*i*	Gajapati	*i*
Parijat	*i*	Satya	*i*	CSR 10	*i*	Pusa 205	*i*
Subhadra	*i*	IRAT 144	*n*	IRBB 4	*i*	IRBB-211	*i*
C102PKT	*i*	IRBLBB-5M	*i*	Pusa 33	*i*	PAU 201	*i*
BL-245	*i*	PR 108	*i*	Suphala	*i*	Ratna	*i*
Badami	*i*	Dhara Heera	*i*	IRBB-8	*i*	PR 115	*j*
Bl-122	*j*	ASD 16	*i*	MI 48	*i*	Ajay	*n*
ADT-36	*i*	A57-115-4	*i*	Jyoti	*i*	Swarna mukhi	*i*
Nialgiri	*i*	IRBLZ5-CA	*i*	IR-8	*i*	NDR 2026	*i*
CO 39	*i*	IRBLB-B	*i*	Pusa 44	*i*	HKR 120	*i*
MAS 868	*i*	TKM-9	*i*	HKR-55	*i*	Sarathi	*i*
Rajendra	*i*	Sadabahar	*i*	Erramallalu	*i*	HUBR-2-1	*i*
Poornima	*i*	Vandana	*i*	PR111	*i*	IRAT 112	*i*
MAS 109	*i*	UPLRI 7	*i*	Pratap	*i*	MTU1010	*i*
Baranideep	*i*	Kranti	*i*	SKR 126	*i*	ADT 37	*i*
Karjat-1	*i*	Saathi	*i*	HUR-4-3	*i*	Pusa Sugandh 5	*i*
IR 50	*i*	Virendra	*i*	PR 114	*i*	HUR-3022	*j*
C101 LAC	*i*	KALYANI 1	*i*	MTU 3626	*i*	PRH 10	*j*
Naggar Dhan	*i*	SKAU 5	*i*	IR 24	*i*	PNR 519	*i*
Deuteshwari	*i*	SKAU 27	*j*	Neela	*i*	Jaya	*i*
VL Dhan 221	*i*	SKAU 382	*i*	PR 113	*i*	Pant Dhan 12	*n*
Khandagiri	*i*	RIL-45	*i*	Keshav	*i*	IC-258220	*i*
Himalaya 799	*i*	CN A 4125	*i*	ADT-38	*i*	VSR 8	*i*
HPR 1068	*i*	VLK 39	*i*	PR 116	*i*	Orugullu	*i*
Pant Dhan 6	*i*	Himdhan	*i*	IR 36	*i*	Karma Mahsuri	*i*
Himalaya1	*i*	CT-10006	*i*	Pathara	*j*	IRBB-203	*n*
ND 118	*i*	CH 45	*i*	Daya	*i*	Tai Pei 309	*i*
Heibao	*i*	PC 19	*i*	Leimphore	*i*	MAS-946	*i*
Palam Dhan 957	*i*	Van Prabha	*i*	IRBL9-W	*i*	ASD 18	*i*
Ananda	*i*	Himalaya2216	*i*	Sebati	*i*	Red Trinaini	*i*
Jogesh	*i*	Samautha	*i*	Samleshwari	*i*	IRBB 21	*i*
Danteshwari	*i*	RP 2421	*n*	Surendra	*i*	PR 120	*i*
IR72	*n*	Kalyani-II	*i*	Kharavada	*i*	CR 2461-9	*i*
Kalinga-II	*i*	SR 1	*i*	Shatabdi	*i*	UPRI-2003-45	*i*
JR 201	*i*	Dular	*n*	Red Triveni	*i*	PNR381	*i*
C101A51(Piz-5)	*j*	IC-248199	*i*	Udayagiri	*i*	Punjab Mehak1	*i*
K 429	*i*	IRBB 5	*i*	NILAGIRI	*i*	PNR 381	*i*
Piz-5	*i*	IRBB 10	*i*	Kharavela	*i*	Jayati	*i*
Karjat-184	*i*	IRBB3	*i*	CR 143-2-2	*i*	Varalu	*i*

Table S1 *(Contd…)*

Genotype	Allelic status	Genotype	Allelic status	Genotype	Allelic status
CT 10006	*i*	Anjali	*i*	Sitwa Dhan	*i*
Sona Khau	*i*	DV 85	*n*	PR118	*i*
Chandrahasini	*n*	1-AC 25	*j*	Gouri	*i*
HPR 2143	*n*	IRAT 240(IREM950)	*i*	Ananga	*i*
MAS 946-1	*i*	CR-2363-26	*j*	Pant dhan 4	*i*
PRR 101	*i*	Selected Sabarmati	*i*	Muskan	*i*
PRR 125	*i*	MTU 1010	*i*	IR 66	*i*
PRR 102	*i*	Kataktara	*n*	HUR-Pm-7m AVTBT	*i*
PRR113	*i*	VOH-PCR-3110	*i*	Abhaya	*i*
PRR 126	*i*	Saanwal Basmati	*i*	OYR 69	*i*
MAS 25	*i*	Lalan Kanda	*n*	ADT 38	*i*
RNRM 7	*i*	C 1268-7-10	*i*	CR 2499	*i*
Pusa Sugandh 2	*i*	BJ-1	*n*	Pusa 1460	*i*
VL7-3	*i*	Vl-95-6-3-9-4	*i*	Sarjoo 52	*i*
VLT-6	*i*	VOH-PCR-3114	*i*	UPRI-2003-24	*i*
Falguna	*i*	CR 2494	*i*	UPRI-2003-18	*i*
Haryana Basmati1	*i*	96718 (CG20)	*i*	Narendra Usar Dhan3	*i*
RIL-10	*i*	BROWN GORA	*n*	NDR 359	*i*
Pant dhan 19	*i*	Tetep Original	*i*	NDR 97	*i*
NDR- 8015-1	*i*	Peeli badam	*n*	Pusa 1176	*n*
Pusa Sugandh 3	*i*	Goal malati	*n*	Bhadrakali	*i*
Pantsankardhan3	*i*	VL-88-97-1-7	*i*	Shiva	*i*
Samanta	*i*	Tripura Medicinal rice	*i*	CR 2364-25	*i*
Tapaswani	*i*	Kamlesh	*n*	IR 77384	*i*
ARS 36	*i*	Hassan serai	*i*	Narendra Usar Dhan2	*i*
HKR-26	*i*	IR-78908	*j*	SKAU 220	*i*
HPR 2083	*i*	Joymati	*n*	PRR 105	*i*
CAN 4136	*i*	Vl-7626	*n*	PRR 111	*i*
Mahamaya	*i*	Abhishek	*n*	PRR 117	*i*
Bhubana	*i*	B6144-MR-6-0-0	*n*	PRR 119	*i*
Pant dhan 10	*i*	Dhoubi	*i*	PRR 103	*i*
BL-142	*i*	PMK-2	*i*	PRR 106	*i*
Sharbati Typical	*i*	VL-9891	*i*	PRR 123	*i*
OYR 128	*n*	Seond Basmati	*i*	PRR 114	*i*
CNA 4130	*i*	HPR 2104	*i*	PRR 120	*i*
OYC 183	*n*	VL-10091	*n*	PRR 124	*i*
Bhanja	*i*	Nagina 22	*n*	PRR 107	*i*
P1463-02-1-1	*i*	Basmati 564	*i*	PRR 115	*i*
BJ-1(Purple awn)	*n*	Sah Pasand	*n*	PRR 104	*i*
Chandana	*i*	Chimbalate Basmati	*i*	PRR 108	*i*
Urvashi	*i*	PMK-1	*i*	PRR 121	*i*
Pant dhan 16	*i*	Dom Siah	*i*	Pant dhan 18	*i*
P1490-03-	*i*	TKM -6	*n*	Indravati	*i*
CSR 27	*i*	Salam pikit	*n*	Pant Sugandh Dhan17	*i*
China 988	*i*	SB-3000	*i*	HUR-200-57-1	*i*

Table S1 *(Contd…)*

Genotype	Allelic status	Genotype	Allelic status	Genotype	Allelic status
Pant dhan 15	*i*	WGL 32100	*i*	Khara Munga	*i*
JGL_11727	*i*	MR-220	*i*	IR 70	*i*
Mahanadi	*i*	Raja Vaellu	*i*	ADT 39	*i*
Sahbhagi Dhan	*i*	CR-246-16	*i*	Haldimuri	*n*
Pant Sugandh Dhan15	*i*	Phunchi	*i*	MTU 7029	*i*
Bhuman San	*i*	Pusa Basmati 1121	*i*	MTU 2067	*i*
JR 75	*i*	Lalat	*i*	PDKV- Chinoor-2	*i*
VOH-PCR-3119	*i*	Improved Sabarmati	*i*	Pusa 1401	*i*
CR-1009	*i*	Tompha Khau	*i*	ON-1	*i*
JR 503	*i*	CN-1268-7	*i*	P-1280-SAF-06	*i*
CO-37	*i*	WGL 14	*i*	Swarna Sub 1	*i*
Sumati	*i*	Hema	*i*	ADT 42	*i*
SAF-1221-83	*i*	UPRVS-98-26	*i*	MTU 5249 (Vajram)	*i*
P 1447	*i*	Pusa 1174	*i*	MTU 4870 (Deepti)	*i*
P1447-00-5-1	*i*	Super Basmati	*i*	Tilak Chandan	*i*
Khao Daen Krai	*n*	Pusa 1301	*i*	Pusa-33	*i*
Khao-Khao	*i*	Kanak	*i*	Mahalaxmi	*i*
Sona Mahsuri	*n*	Indira sona	*i*	Meher	*i*
IC-86318	*i*	PRR 109	*i*	Pusa Basmati 1	*i*
IRBB 54	*i*	PRR 110	*i*	Pratikshya	*i*
CSR 13	*i*	PRR 127	*i*	Jhulhat	*i*
HUR-105	*i*	PRR 116	*i*	NDR 9830144	*i*
Raskandam	*i*	PRR 122	*i*	MTU 1001 (Vijetha)	*i*
HUR 105	*i*	PRR 118	*i*	Santepheap	*i*
Indira Sugandh Dhan1	*i*	HUR-36	*i*	Prachi	*n*
Manaswini	*n*	Kalinga-III	*i*	Uphar	*i*
RAU3002	*i*	RR 166-645	*n*	Dubraj	*i*
RR 8585	*i*	NDR- 8011	*i*	Ramachandi	*i*
CSR 23	*i*	GSR-2-1-46	*i*	Jagabandhu	*i*
Kudrat-3	*i*	Malviya Dhan	*i*	4010	*i*
RAU 3061	*i*	C 22	*i*	CRRI Black Aroma	*i*
PR 600	*i*	DHMAS-70G-16429	*i*	CSR 36	*i*
CN-1793	*i*	HKR-39	*i*	Acharmati	*n*
NDR 625	*i*	Ranbir Basmati	*i*	Chinikamini	*n*
Sagar dambha	*i*	T23	*i*	Singhuva Malet	*i*
JGL-3828	*i*	Jeeraga Sambha	*i*	NDR 9830135	*i*
P1460	*i*	ASD 19	*i*	Intan	*i*
Pusa1342	*i*	Basmati 370	*i*	NDKN-3327 (SN)	*i*
MR-219	*i*	Zang Bhuman	*i*	Taraori Basmati	*i*
UPRI-2003-15	*i*	IC-2127	*i*	TMK-6	*i*
Sambha Mahsuri	*i*	VOH-PCR-3113	*i*	Rambha	*i*
Sharbati	*i*	TYPE-3	*i*	Kalanamak-3119	*i*
HKR-200-57-1	*i*	Nagina 12	*i*	Basmati 386	*i*
WGL-23985	*i*	Sonasal	*i*	VOH-PCR-3139	*i*
Rajendra basmati	*i*	Bhuman Nagau	*i*	Gaur1	*i*

Table S1 *(Contd…)*

Genotype	Allelic status	Genotype	Allelic status	Genotype	Allelic status
Gaur2	*i*	GR3	*i*	Gar13	*i*
Gaur10	*i*	GR4	*i*	GR101	*i*
Gaur100	*i*	GR5	*n*	GR102	*i*
Gar1	*i*	GR6	*i*	GR103	*i*
Gar2	*i*	GR7	*i*	GR104	*i*
GR11	*n*	GR8	*n*	Gurjari	*i*
GR12	*i*	GR9	*n*	SK20	*i*
J280	*i*	Dandi	*i*	Mahsuri	*i*
P Basmati	*i*				

Where *n-neutral* allele (*S5-n*), *i* - *indica* allele (*S5-i*) and *j* – *japonica* allele (*S5-j*)

Table S2. List of primers used for screening of the rice germplasm

Primer name	LG	Target	Sequence	Tm (^{0}C)	Product size		
					i	***j***	***n***
S5-InDel	6	*S5-n*	F-CCTACGTTTGACTGCCTGCCTG	61.0	417bp	417bp	281bp
			R-CTACACGCGGCTTCGGGAAAGC	63.4			
S5-ELSP1	6	*S5-i*	F-GACAGCAGCATCAACGACTTCC	59.1	527bp	No. Amp	527bp
S5-IASP2			R-TCGTCAGTGGGCAAGCAGTAGCTG	63.3			
S5-JASP1	6	*S5-j*	F-ACCCTGATATTCTGAGTTACAAGGCATTA	57.4	No. Amp	325bp	No. Amp
S5-ELSP2			R-GCTCTTGATGTCCGGTGATACC				

ELSP, external locus specific primer; IASP, *indica* allele specific primer; JASP, *japonica* allele specific primer

Table S3: List of wide compatible varieties identified

Genotypes	Genotypes	Genotypes	Genotypes
IRGC7486	TPJ 210	IRG-144	Chandrahasini
IRGC1797	TPJ 218	IRG-150	HPR 2143
IRGC5320	TPJ 229	IRG-164	OYR 128
IRGC3849	TPJ 232	IRG-165	OYC 183
IRGC6457	TPJ 233	IRG-167	BJ-1(Purple awn)
IRGC5441	TPJ 238	IRG-169	DV 85
IRGC5097	TPJ 245	IRG-173	Kataktara
IRGC4122	TPJ 247	IRG-179	Lalan Kanda
IRGC8261	TPJ 250	IRG-181	BJ-1
IRGC10758	IRG-15	IRG-185	BROWN GORA
IRGC11336	IRG-21	IRG-200	Peeli badam
IRGC14738	IRG-22	IRG-204	Goal malati
IRGC15006	IRG-40	IRG-219	Kamlesh
IRGC10658	IRG-47	IRG-239	Joymati
IRGC8182	IRG-55	IRG-242	Vl-7626
IRGC5726	IRG-56	IRG-243	Abhishek
IRGC137	IRG-61	IRG-244	B6144-MR-6-0-0
IRGC6937	IRG-62	IRG-245	VL-10091
IRGC1972	IRG-64	IRG-247	Nagina 22
IRGC1739	IRG-65	IRG-249	Sah Pasand
IRGC17906	IRG-67	IRG-251	TKM -6
IRGC18936	IRG-75	IRG-255	Salam pikit
IRGC64911	IRG-79	IRG-258	Pusa 1176
IRGC71559	IRG-82	IRG-271	Khao Daen Krai
IRGC69911	IRG-92	IRG-298	Sona Mahsuri
IRGC25660	IRG-98	IRG-311	Manaswini
IRGC66758	IRG-107	IRG-324	RR 166-645
IRGC66529	IRG-110	IRG-326	Haldimuri
IRGC50399	IRG-112	IRG-333	Prachi
IRGC38994	IRG-115	IRG-337	Acharmati
IRGC32406	IRG-116	IRG-341	Chinikamini
IRGC32388	IRG-121	IRG-353	GR5
IRGC31051	IRG-125	IRG-361	GR8
IRGC30921	IRG-127	IRG-374	GR9
IRGC24528	IRG-128	IR72	GR11
IRGC6741	IRG-133	IRAT 144	
IRGC24274	IRG-137	RP 2421	
IRGC55808	IRG-139	Dular	
TPJ 203	IRG-141	Ajay	
TPJ 205	IRG-142	PantDhan12	
TPJ 206	IRG-143	IRBB-203	

Table S4: Mean of hybrids for spikelet fertility (%) in *indica* and *japonica* subgroups

**Groups**	**Count**	**Mean of spikelet fertility (%)**			
		**Aduthurai**	**Cuttack**	**Barapani**	**Overall**
i **x** ***n***	82	69.00	74.00	72.46	71.81
j **x** ***n***	82	71.30	72.83	72.69	72.27
**Total**	164	70.14	73.36	72.57	72.04

Where *n* - *neutral* allele (*S5-n*), *i* - *indica* allele (*S5-i*) and *j* – *japonica* allele (*S5-j*)

Table S5: Mean performance of hybrids across three locations

Cross combination	Genotypes	PH (cm)	NT	PL	FG	UFG	SF (%)	YPP (gm)
Pusa 44*IRG-337	WCH1	140.1	28.5	4.9	94.7	39.2	70.7	24.7
Pusa 44*IRG 2421	WCH2	122.0	20.2	6.2	135.7	14.6	90.3	21.7
Pusa 44*IRG-141	WCH3	114.7	25.1	9.7	84.4	27.0	77.5	29.9
Pusa 44*Joymati	WCH4	117.6	23.0	7.1	127.2	33.8	79.1	35.4
Pusa44*Kamlesh	WCH5	108.2	18.4	8.1	87.8	49.8	62.6	17.8
Pusa 44*TPJ 206	WCH6	142.4	29.2	10.8	154.7	122.3	55.7	20.4
Pusa 44*IRGC32406	WCH7	112.0	26.2	4.3	113.4	78.2	59.0	23.9
Pusa 44*IRGC6937	WCH8	121.9	24.9	11.6	143.2	54.7	70.3	34.6
Pusa 44*Nagina 22	WCH9	112.7	24.3	7.6	110.1	27.6	79.6	16.1
Pusa 44*GR8	WCH10	121.7	22.0	10.6	103.1	20.2	85.4	37.2
Pusa 44*IRG-47	WCH11	106.7	20.9	12.7	60.4	24.1	71.0	10.7
Pusa 44*IRGC137	WCH12	126.2	17.1	18.1	154.5	102.5	60.2	22.6
Pusa 44*IRG-179	WCH13	104.2	21.3	10.3	40.1	36.3	52.6	12.4
Pusa 44*BJ-1(Purple awn)	WCH14	151.9	29.1	5.0	163.5	72.9	71.9	26.2
Pusa 44*Kataktara	WCH15	119.4	22.5	6.6	102.3	12.6	89.0	27.2
Pusa 44*Dular	WCH16	107.4	24.2	8.1	95.0	14.0	88.0	27.3
Pusa 44*TPJ 245	WCH17	101.6	19.7	9.6	106.0	18.1	86.5	30.7
Pusa 44*IRG-144	WCH18	111.3	18.9	9.3	81.6	39.7	70.4	11.7
Pusa 44*IRGC11336	WCH19	141.6	30.8	5.7	93.2	74.0	57.7	34.3
Pusa 44*IRG-55	WCH20	114.5	22.2	7.2	104.0	12.2	89.5	15.3
Pusa 44*OYC 183	WCH21	113.4	22.4	8.1	98.7	23.2	82.8	31.1
Pusa 44*IRG-142	WCH22	115.8	27.0	6.3	118.0	22.7	84.1	36.6
Pusa 44*IRG-143	WCH23	121.5	26.0	10.3	106.1	15.8	86.9	47.1
Pusa 44*IRG-204	WCH24	109.6	21.7	8.3	79.0	30.0	75.1	34.9
Pusa 44*OYR 128	WCH25	122.9	19.2	21.1	159.8	93.6	63.1	40.4
Pusa 44*IRGC50399	WCH26	118.5	24.0	9.9	72.2	31.6	69.7	22.1
Pusa 44*PR 120	WCH27	124.1	24.1	10.0	60.6	40.8	62.7	24.8
Pusa 44*IRGC5441	WCH28	124.3	22.9	8.7	63.7	32.2	66.8	19.1
Pusa 44*IRG-137	WCH29	138.9	21.7	22.2	130.2	32.9	80.8	29.5
Pusa 44*IRG-164	WCH30	119.3	21.8	5.9	46.6	43.5	54.4	12.2
Pusa 44*DV 85	WCH31	143.1	20.5	24.1	92.2	21.7	80.9	34.4
Pusa 44*IRGC8182	WCH32	99.0	19.8	5.3	58.7	44.6	61.6	10.1
Pusa 44*IRG-169	WCH33	91.7	23.6	17.4	136.3	44.4	78.4	11.8
Pusa 44*IRGC71559	WCH34	112.7	23.0	10.1	123.1	40.8	75.0	18.8
Pusa 44*Peeli badam	WCH35	123.8	22.5	8.6	123.2	19.0	87.3	32.0
Pusa 44*Lalan Kanda	WCH36	117.6	24.4	10.1	156.9	27.2	85.6	29.4
Pusa 44*IRG-200	WCH37	126.7	26.7	12.2	111.3	31.9	77.7	31.2
Pusa 44*TPJ 247	WCH38	117.2	19.7	8.1	73.3	20.0	78.4	11.9
Pusa 44*IRGC5726	WCH39	109.0	24.3	8.8	125.9	32.5	80.1	12.4
Pusa 44*IRG-251	WCH40	124.5	21.2	7.5	151.1	42.6	78.6	39.1
Pusa 44*IRGC10658	WCH41	127.2	26.5	7.1	192.2	42.1	83.7	37.2
IR64*IRGC50399	WCH42	120.3	21.0	8.7	66.0	36.7	64.5	19.1
IR64*PR 120	WCH43	113.4	21.6	9.0	105.7	26.1	81.3	9.8
IR64*IRG-143	WCH44	110.0	21.8	7.9	65.2	26.7	74.6	17.6
IR64*IRGC8182	WCH45	102.4	24.4	12.0	34.5	29.8	54.0	4.7
IR64*Peeli badam	WCH46	108.4	21.1	7.3	106.6	24.3	82.3	16.7
IR64*Kataktara	WCH47	109.8	27.0	6.2	93.7	11.5	89.4	18.4
IR64*OYR 128	WCH48	98.0	22.5	7.1	57.0	42.9	56.8	11.1
IR64*Lalan Kanda	WCH49	110.8	24.4	6.8	72.3	48.4	63.2	39.9

Supplementary Table S5 (*Contd…)*

Cross combination	Genotypes	PH (cm)	NT	PL	FG	UFG	SF (%)	YPP (gm)
IR64*IRG-337	WCH50	135.3	26.2	5.9	94.6	33.8	73.6	24.5
IR64*TPJ 206	WCH51	135.5	26.6	10.5	151.3	106.5	58.8	24.5
IR64*IRG-204	WCH52	110.6	19.0	10.9	86.6	36.6	73.3	31.6
IR64*IRG-47	WCH53	101.4	23.2	6.6	55.9	38.9	58.2	11.7
IR64*TPJ 245	WCH54	98.2	23.4	8.7	114.3	17.7	87.8	30.3
IR64*RP 2421	WCH55	99.9	20.8	7.0	125.4	26.8	82.9	35.0
IR64*IRGC32406	WCH56	119.9	23.8	5.7	95.3	13.7	87.3	17.6
IR64*IRG-251	WCH57	118.8	22.2	9.3	54.8	41.5	57.6	15.2
IR64*IRG-251	WCH58	107.8	25.4	10.2	122.2	20.0	86.6	40.5
IR64*IRG-137	WCH59	122.3	23.2	9.2	154.1	21.2	88.2	16.1
IR64*IRGC5441	WCH60	102.3	24.6	7.5	54.3	41.4	60.2	25.8
IR64*IRG-144	WCH61	113.1	21.7	7.5	81.6	41.7	69.5	12.8
IR64*IRG-169	WCH62	103.5	19.7	22.3	64.7	66.7	49.3	13.8
IR64*Joymati	WCH63	118.9	24.3	20.1	80.1	80.9	49.7	20.4
IR64*IRGC6937	WCH64	129.0	18.7	16.7	69.1	73.3	48.4	21.1
IR64*GR8	WCH65	100.2	20.7	7.1	75.4	41.9	65.0	16.9
IR64*TPJ 247	WCH66	123.7	25.1	7.9	21.3	74.9	22.2	14.8
IR64*Dular	WCH67	106.7	22.2	7.2	55.0	50.4	52.4	13.8
IR64*IRGC10658	WCH68	117.1	24.1	6.8	94.0	19.2	83.0	15.6
IR64*IRG-55	WCH69	112.4	22.6	7.1	48.9	25.9	66.7	11.3
IR64*IRG-164	WCH70	105.4	23.2	6.9	54.8	29.7	64.8	10.5
IR64*OYC 183	WCH71	104.3	23.4	9.2	43.2	29.7	59.3	11.0
IR64*DV 85	WCH72	100.3	24.8	9.0	68.8	23.1	74.8	21.8
IR64*IRGC137	WCH73	97.6	24.0	8.1	69.1	14.3	83.4	17.5
IR64*IRGC11336	WCH74	126.1	26.9	7.1	55.6	49.6	52.8	11.3
IR64*Kamlesh	WCH75	118.6	22.7	11.7	96.2	34.6	73.6	10.1
IR64*IRG-200	WCH76	105.5	22.7	6.9	53.3	19.2	73.3	9.6
IR64*IRG-141	WCH77	106.6	22.2	8.2	67.0	6.1	91.5	11.7
IR64*IRG-179	WCH78	104.7	24.9	8.6	64.6	27.4	70.1	11.9
IR64*IRG-142	WCH79	122.6	25.4	8.8	75.5	16.3	82.2	11.6
IR64*IRGC5726	WCH80	107.8	26.9	5.3	155.8	34.8	82.3	14.1
IR64*IRGC71559	WCH81	116.7	20.3	8.9	117.7	37.3	76.0	17.7
IR64*Nagina22	WCH82	99.7	21.3	7.7	36.3	26.9	56.7	9.5
IRGC8146 *OYC 183	WCH83	113.1	24.9	9.9	165.7	54.3	75.7	28.0
IRGC8146*OYR 128	WCH84	118.3	24.4	8.4	146.4	34.7	81.6	48.7
IRGC8146*IRGC50399	WCH85	112.8	24.0	4.3	64.6	22.0	73.4	6.7
IRGC8146*IRGC5441	WCH86	118.7	24.8	6.8	142.7	26.3	85.4	27.4
IRGC8146*IRG-251	WCH87	141.0	28.2	5.6	76.9	49.9	60.7	20.5
IRGC8146*PR 120	WCH88	106.9	24.5	8.4	52.9	13.6	80.4	12.0
IRGC8146*IRG-137	WCH89	127.3	25.3	6.2	81.2	9.0	90.0	19.0
IRGC8146*IRG-141	WCH90	107.6	22.0	8.1	61.3	16.9	79.8	24.0
IRGC8146*IRG-169	WCH91	99.8	23.2	6.5	75.0	17.0	81.5	17.5

Supplementary Table S5 (*Contd…)*

Cross combination	Genotypes	PH (cm)	NT	PL	FG	UFG	SF (%)	YPP (gm)
IRGC8146*IRGC8182	WCH92	103.6	21.0	7.4	110.8	35.0	76.4	15.6
IRGC8146*DV 85	WCH93	120.7	22.9	7.8	120.6	27.6	82.2	32.0
IRGC8146*IRG-251	WCH94	147.1	21.3	21.5	29.1	132.6	17.6	8.3
IRGC8146*IRG-142	WCH95	133.5	25.5	10.0	59.1	66.7	45.7	17.6
IRGC8146*Dular	WCH96	108.0	24.0	7.4	66.7	9.0	88.1	15.5
IRGC8146*Lalan Kanda	WCH97	118.0	22.8	8.9	176.1	56.2	76.0	25.3
IRGC8146*IRG-204	WCH98	109.5	21.9	12.2	115.4	50.8	69.6	26.4
IRGC8146*Nagina22	WCH99	115.0	20.8	6.7	53.1	34.1	61.3	23.8
IRGC8146*Kataktara	WCH100	127.2	24.0	8.3	78.5	16.2	82.1	23.5
IRGC8146*IRG-179	WCH101	119.1	19.6	9.7	61.5	21.7	74.4	28.0
IRGC8146*IRG-143	WCH102	124.2	27.1	8.7	100.5	105.0	48.9	20.5
IRGC8146*IRGC71559	WCH103	118.3	21.7	8.3	96.1	22.7	81.4	22.0
IRGC8146*IRG-55	WCH104	124.8	25.0	8.8	86.4	22.0	81.0	18.2
IRGC8146*IRGC5726	WCH105	112.2	22.6	8.0	63.1	83.1	43.0	15.5
IRGC8146*IRGC32406	WCH106	103.4	18.8	9.3	121.8	67.6	64.5	25.0
IRGC8146*IRG-164	WCH107	128.6	20.0	12.8	85.5	19.5	81.5	19.2
IRGC8146*Joymati	WCH108	107.5	24.6	12.2	98.4	27.4	78.5	35.2
IRGC8146*IRG-337	WCH109	134.0	26.9	6.1	108.6	89.6	54.8	14.0
IRGC8146*IRG-144	WCH110	108.6	18.6	8.5	82.2	38.3	68.4	17.2
IRGC8146*RP 2421	WCH111	107.1	22.6	7.5	83.8	71.7	54.5	31.7
IRGC8146*Kamlesh	WCH112	99.8	26.2	7.4	64.8	31.0	70.0	29.5
IRGC8146*TPJ 206	WCH113	148.0	27.6	5.2	98.9	87.9	53.0	11.3
IRGC8146*IRGC10658	WCH114	123.1	26.0	7.7	59.4	51.1	59.2	13.3
IRGC8146*Peeli badam	WCH115	130.2	21.6	7.2	90.2	42.4	69.7	27.7
IRGC8146*GR8	WCH116	112.7	24.1	6.6	67.6	45.8	59.7	25.0
IRGC8146*IRG-47	WCH117	136.3	24.4	6.6	136.0	17.6	88.6	12.9
IRGC8146*TPJ 247	WCH118	123.8	24.2	4.2	85.3	20.7	80.5	8.2
IRGC8146*TPJ 245	WCH119	117.8	24.6	8.3	130.0	34.7	79.5	27.7
IRGC8146*IRGC137	WCH120	129.6	20.2	12.9	188.3	41.2	82.2	32.4
IRGC8146*IRGC11336	WCH121	141.2	20.2	13.9	158.9	63.4	71.6	38.3
IRGC8146*IRG-200	WCH122	114.9	21.2	21.3	129.0	26.3	83.4	24.3
IRGC8146*IRGC6937	WCH123	134.3	28.9	6.0	106.7	66.5	61.6	14.4
IRGC15046*OYR 128	WCH124	127.3	18.8	12.8	129.2	31.8	80.3	19.9
IRGC15046*IRGC10658	WCH125	122.3	21.7	4.6	98.2	20.1	84.4	46.6
IRGC15046*IRGC5441	WCH126	121.8	23.2	7.6	74.6	14.4	84.8	14.7
IRGC15046*IRG-251	WCH127	132.9	25.7	8.4	62.3	15.3	80.8	15.3
IRGC15046*PR 120	WCH128	113.5	20.0	19.6	65.1	11.1	85.2	13.6
IRGC15046*IRG-137	WCH129	140.1	24.6	7.4	88.1	27.1	78.7	53.5
IRGC15046*IRG-141	WCH130	118.9	24.2	12.4	85.4	24.7	79.2	22.0
IRGC15046*IRG-169	WCH131	101.5	24.8	9.4	68.0	24.4	73.4	25.4
IRGC15046*IRGC8182	WCH132	111.1	23.3	9.2	62.5	29.6	68.2	8.6
IRGC15046*DV 85	WCH133	120.6	27.1	9.9	56.6	42.2	57.5	32.5

Supplementary Table S5 (*Contd…)*

Cross combination	Genotypes	PH (cm)	NT	PL	FG	UFG	SF (%)	YPP (gm)
IRGC15046*IRG-251	WCH134	129.7	29.7	9.3	51.0	152.5	25.1	9.9
IRGC15046*IRG-337	WCH135	129.2	20.9	6.1	113.6	80.3	58.6	17.3
IRGC15046*Kamlesh	WCH136	137.2	22.9	8.5	77.5	26.6	75.6	31.0
IRGC15046*Lalan Knda	WCH137	146.1	19.6	19.2	160.9	49.9	76.7	26.5
IRGC15046*IRG-204	WCH138	115.1	28.1	25.2	85.7	31.3	73.2	32.7
IRGC15046*Nagina22	WCH139	105.3	19.8	9.6	136.5	40.7	77.3	24.7
IRGC15046*Kataktara	WCH140	117.1	25.2	7.0	82.0	30.3	73.1	29.0
IRGC15046*IRG-179	WCH141	94.7	19.3	8.1	62.1	28.6	72.2	22.6
IRGC15046*IRG-47	WCH142	125.6	19.3	8.5	124.0	18.3	87.0	13.6
IRGC15046*IRG-200	WCH143	120.2	19.8	20.5	137.0	18.0	88.4	28.3
IRGC15046*IRGC71559	WCH144	113.2	23.2	6.7	106.2	36.8	77.4	11.1
IRGC15046*Dular	WCH145	123.2	23.6	9.2	77.5	12.6	86.0	13.9
IRGC15046*IRG-142	WCH146	119.2	20.1	11.8	69.3	52.9	56.3	12.1
IRGC15046*IRGC5726	WCH147	125.2	20.9	16.5	91.7	28.0	76.7	18.4
IRGC15046*IRG-164	WCH148	124.5	18.3	12.0	83.6	17.9	82.6	19.9
IRGC15046*WCV86	WCH149	124.8	19.1	24.7	97.0	37.8	72.0	29.8
IRGC15046*Pili badam	WCH150	127.6	20.5	10.7	87.9	39.2	70.2	18.4
IRGC15046*Joymati	WCH151	135.4	22.8	8.1	182.0	71.7	71.8	10.5
IRGC15046*IRG-144	WCH152	120.6	21.5	18.2	177.8	39.0	82.1	25.1
IRGC15046*RP 2421	WCH153	124.9	20.5	18.0	101.2	58.8	63.3	19.1
IRGC15046*TPJ 206	WCH154	112.0	25.0	8.4	62.5	28.7	68.5	13.4
IRGC15046*IRGC137	WCH155	136.2	20.5	15.6	189.8	31.3	85.8	35.2
IRGC15046*IRG-55	WCH156	116.2	22.2	11.5	80.3	18.9	80.9	14.0
IRGC15046*GR8	WCH157	109.6	21.9	6.7	77.8	30.5	76.2	31.1
IRGC15046*TPJ 247	WCH158	129.4	23.5	5.3	62.3	20.7	75.3	15.9
IRGC15046*TPJ 245	WCH159	97.9	19.9	7.1	140.2	35.6	79.8	12.1
IRGC15046*IRGC11336	WCH160	147.1	19.0	14.6	177.7	78.8	69.3	41.3
IRGC15046*IRG-143	WCH161	123.2	23.2	10.5	107.9	95.3	53.1	21.7
IRGC15046*IRGC50399	WCH162	114.7	20.3	7.9	79.7	17.0	82.4	11.3
IRGC15046*IRGC6937	WCH163	123.8	24.7	4.0	109.8	63.0	63.6	17.1
IRGC15046*OYC 183	WCH164	116.0	20.6	10.6	155.0	48.7	76.4	27.1
Pusa 44	Check1	124.0	21.5	20.2	137.3	56.3	70.9	30.3
IR64	Check2	120.3	20.5	20.5	99.0	61.0	61.9	31.6
IRGC8146	Check3	105.3	23.9	19.3	128.3	35.1	78.5	31.1
IRGC15046	Check4	103.3	23.2	20.8	110.3	18.3	85.8	28.3
	**Minimum**	91.7	17.1	4.0	21.3	6.1	17.6	4.7
	**Maximum**	151.9	30.8	25.2	192.2	152.5	91.5	53.5
	**Mean**	118.1	23.0	10.0	96.9	39.3	72.1	22.0
